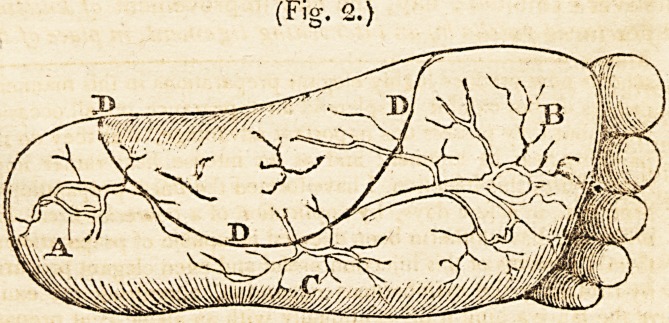# Observations on the Integuments of the Human Body

**Published:** 1814-06

**Authors:** Alex. Ramsay

**Affiliations:** 3, Villa Place, Walworth Common, near London


*For the Medical and Physical Journal.*

### *Observations on the Integuments of the Human Body.*

By Alex. Ramsay, M.D.

FOR a long series of years, my attention has been directed to the structure, offices, and phenomena of the integuments of the human species—I am sorry to say with very little success, respecting practical improvement in the treatment of their diseases.—In my schools in Europe, and abroad, I have been enabled to investigate these organs in subjects of of most civilised nations. I shall consider this subject under the regular order in which the parts present themselves to our attention, in tracing their various appendages, beginning at the transmission of the vessels from the muscles.

*Vessels of the Integuments.*

(Fig. 1.)

The diagram, Fig. 1. conveys an exact delineation of the powerful vascularity of this organ in North America, from a preparation formed for the purpose.* This was taken from part of the palmar integuments. The more soft portions of the

* I find that the learned and ingenious Mr. Charles Bell, of London, has given the description of the injection I discovered when a pupil with Dr. Marshal, of Thavies Inn, London, and which I inserted in the New-York Repository, (when I visited that metropolis in 1803,) edited by that very able physician, Dr. Mitchell, of New York. The injection I use is the following:—Take of white or red oxyde of lead, such a quantity as may be wanted; add to this oxyde as much water as admits of the oxyde uniting with raw lintseed-oil; put no more oil to the preparation than is needed to form a thick paste; levigate this in the most minute manner in a mortar, or on a tile or stone; beat up or mix this (in as rapid a manner as possible, as it often becomes hardened in a minute,) with a varnish composed of equal weights of resin and spirit of turpentine, so as to render the fluid as thin as cream. Throw the injection immediately into the preparation. Wherever pressure is suspected, endeavour to counteract this by some means or other. Thus, if you inject the aorta with the intestines and lungs insitu, turn the subject on the face till the injection is hardened, which often is complete in a quarter of an hour. I must warn practitioners, that these oxydes soon acquire a black colour, the preparation must not be long in hand. The American schools the system exhibited still higher degrees of profusion and enlargement, especially the leg, in consequence of the people wearing boots in those ardent heats, which I believe a very deleterious practice in all climates, though now prevalent in many civilised countries.—A B C D, are intended to represent the vessels and corpus adiposum. I, the cutis vera. A, a common arterial trunk, sending off B B rami. C, an arterial circle, sending off E, a perspiring artery.* D, represents an artery (springing from the arterial circle C,) diffused on the cells of the corpus adiposum. E, the artery, penetrating the cutis vera, arises from the same circle. F G H, form similar arterial distributions.

### *Observations on the Vascular Economy.*

Many authors have written so ably on the subject of cutaneous diseases, that I cannot add any remark of use to the public. There is one circumstance, however, I beg leave to mention, that affections of the vessels of the scalp seem very common, not only in cases of poverty, but likewise in cases of excessive luxury. Practitioners seem too frequently to depend on external applications on such occasions, in place of resorting to feeding the poor, and prescribing temperance to the rich. I have been long induced to believe, that vessels receive, *ab origine*, that structure from which peculiar action and depositions have their source; when these become once altered, they do not again readily resume their former structure or action, and *producing powers*, if I may take the liberty of using this expression. Thus in bone, which presents us with more unequivocal instances than any other substance, whenever its vessels are once disturbed by the process of anchylosis, exostosis, or callus by previous fracture, the cancellated or regular structure is never exhibited; nay, the new improvement *of knitting the fractured patella by an intervening ligament*, *in place of bring-*

schools now produce highly elegant preparations in this manner. The colours do not exhibit so splendid an appearance on all occasions as wax, but they possess this important advantage, that they do not expand by heat in summer; and as an intense heat rather improves than injures this injection, I have formed the finest preparations of an arm, &c. in a few days, by application of a powerful fire. Hearts, when fat, have hitherto been deemed incapable of preparation; they form by means of this injection useful and even elegant preparations; by rupturing the membranous expansion, permitting the exudation of the fat, washing it off continually with an alkalescent preparation, a heart may be dried in a few days.

* I have exhibited the appearance of seemingly real effusing arteries occasionally.

*ing*

*ing the fractured portions in contact*, *as done in former times*, derives its preference from the brittle texture of the osseous matter deposited, when the bones are brought nearly into contact. The observations I had an opportunity of making on the black species of our race abroad, confirmed me in the belief, that this remark of vessels never resuming their original character when once essentially disturbed, reaches to the vessels in every part of the system, especially those of the cutis vera, which are the terminations of those of the corpus adiposum. When the cutis vera is fairly and completely destroyed, its vessels never seem regenerated, nor the substance of the cutis vera reproduced; hence, the filmy, unorganised, glazed appearance of the skin, the black membrana mucosa is wanting, the skin appearing perfectly white. These cases from which I drew my inferences, arose from burning, scalds, &c. which had taken place years before.

*Deductions drawn from the Vascular Distributions of the Integuments.*

May we deduce practical opinions, respecting the causes of obesity, emaciation, &c. from the peculiar distribution and anastomoses of the vessels of the integuments?—If E, Fig. 1, admits of perspiration, it would seem that there will be the less return, *by direct continuity of canal of the circle* C, to the adipose vessels D, hence obesity will be diminished, *vice versâ*; hence, too, may we account for the retention of caloric and its evaporation, as well as the peculiar phenomena of sensations in the warm and cold paroxysm of fever? May we suppose, that by whatever means perspiration is diminished, *by debility giving rise to spasmodic contraction*, *or by impervious organic structure of the integuments*, adeps will be augmented?—Thus, in the sole of the foot, Fig. 2,

(Fig. 2.)

A B C, *the heel*, *outer margin of the foot*, *and the anterior terminations of the metatarsus*, where the cuticula is accumulated in numerous laminæ, by compression, the impervious

 state state of these parts occasions the deposition of adipose matter; here, likewise, vesication takes place, when by exercise there is more deposition than return of serous effusion, which has no means of escape but by the dead lamina being thrown off.

I have represented, in Fig. 2, those circular arterial anastomoses at A, B, and C, which keep up continual connection between the adipose and perspiring system of vessels. The circular line D D D represents the hollow of the foot, where the integuments are pervious; hence perspiration is carried on uninterruptedly, and here vesication seldom occurs.

May we, on the same principle, suppose, that great debility, terminating in great relaxation, may induce emaciation and universal imbecility, exhibiting excessive perspiration, often however attended by adeps in abundance, approximating dropsy?—Thus, if I am correct in my first fasciculus, *honoured by your animadversions*, all maladies seem the consequence of debility; the muscular annuli of the vessels assuming either spasmodic contraction or excessive relaxation, approximating dissolution, which seem contrasted, and often alternating extremes of legitimate structure and action. The general imbecility of such cases does not arise wholly from perspiration, but perspiration seems an effect of that feebleness which reigns throughout the whole system of the heart and arteries. Hence in fever, perspiration is not the cause of cure, but the effect of stimulation applied, for the patient is not convalescent till perspiration ceases.

In such subjects, the muscles of the arteries are scarcely perceptible in some cases; the muscles of the heart and limbs are pale, soft, often interlarded with fat, and quickly suffer putrescence, &c.

May we suppose, that the integuments form a most important vital organ? and their vessels effuse, *through the medium of moisture or serum*, superabundant caloric, accumulated in violent exertions?—Do the passions produce violent affections of the heart, terminating in the peculiar affections of their vessels?—Certain notes of music promote a peculiar thrill and chilliness, especially on the covered parts of the frame, the anterior surfaces of the thigh, &c. so hatred, fear, love, seem to promote the same affections. Are these ultimate consequences of intellection?

Permit me to finish this essay, in requesting of your learned readers to favour me with their opinion, what can be the cause of those injuries of the integuments of the horse species, which commonly occasion an alteration of colour from from dark to white? Is it owing to the destruction of the rete mucosum?

Some of the best physiologists I have met with have been at a loss to account for the palm, and sole of the foot, where the black treads, being as white as those of our species? The anatomist will find, I suppose, that it is owing to the numerous lamina of the cuticula, which eclipses the rete mucosum. ALEX. RAMSAY, M.D.

*3, Villa Place, Walworth Common,*
*near London.*

## Figures and Tables

**Fig. 1. f1:**
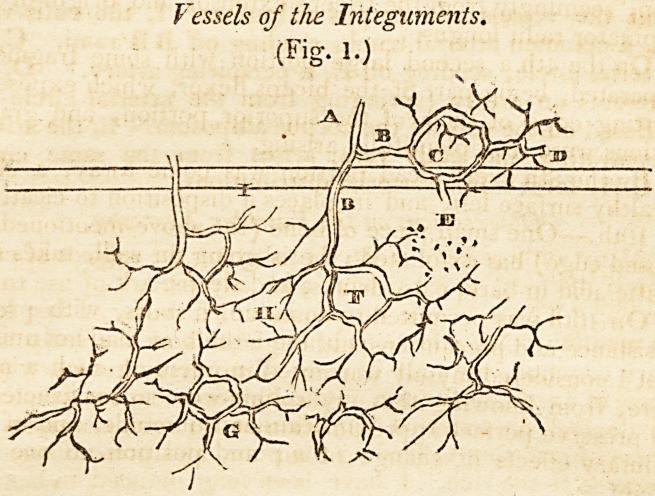


**Fig. 2. f2:**